# Interleukin-6 signalling in health and disease

**DOI:** 10.12688/f1000research.26058.1

**Published:** 2020-08-20

**Authors:** Stefan Rose-John

**Affiliations:** 1 Biochemical Institute, Christian-Albrechts-Universitaet zu Kiel, Olshausenstrasse 40, D24098 Kiel, Germany

**Keywords:** gp130, sgp130Fc, IL-6, IL-6R, sIL-6R, trans-signalling, ADAM17

## Abstract

Biochemically, interleukin-6 belongs to the class of four-helical cytokines. The cytokine can be synthesised and secreted by many cells. It acts via a cell surface-expressed interleukin-6 receptor, which is not signalling competent. This receptor, when complexed with interleukin-6, associates with the signalling receptor glycoprotein 130 kDa (gp130), which becomes dimerised and initiates intracellular signalling via the Janus kinase/signal transducer and activator of transcription and rat sarcoma proto oncogene/mitogen-activated protein kinase/phosphoinositide-3 kinase pathways. Physiologically, interleukin-6 is involved in the regulation of haematopoiesis and the coordination of the innate and acquired immune systems. Additionally, interleukin-6 plays an important role in the regulation of metabolism, in neural development and survival, and in the development and maintenance of various cancers. Although interleukin-6 is mostly regarded as a pro-inflammatory cytokine, there are numerous examples of protective and regenerative functions of this cytokine. This review will explain the molecular mechanisms of the, in part opposing, activities of the cytokine interleukin-6.

## Introduction

Interleukin-6 (IL-6) is considered one of the most prominent pro-inflammatory cytokines ^1^. Blockade of IL-6 by the neutralising monoclonal antibody tocilizumab has been approved in more than 100 countries for the treatment of patients with autoimmune disorders such as rheumatoid arthritis ^2^. Additionally, the cytokine storm sometimes encountered when cancer patients are treated with chimeric antigen receptor (CAR) T-cells ^3^ could be effectively treated with the antibody tocilizumab, leading to US Food and Drug Administration (FDA) approval of the drug for this condition. Even more recently, it has been recognised that many patients experience a similar cytokine storm upon infection with SARS-CoV-2 (COVID-19) virus ^4^ and that these patients could also be treated with tocilizumab ^5^. These new data led to a rekindled general interest in the cytokine IL-6.

IL-6 was initially discovered and cloned in the Kishimoto laboratory as a B-cell stimulatory factor ^6^. Immediately after the molecular cloning, it was evident that IL-6 was identical to hepatocyte stimulating factor ^7^, hybridoma-plasmacytoma growth factor ^8^, interferon β2 ^9^, and 26 kDa protein ^10^. This already indicated the pleiotropic nature of the cytokine. Later on, it was also recognised that IL-6 shows profound activities in the brain ^11, 12^, in the regulation of metabolism ^13, 14^, in the response of the body to exercise ^15^, and in the development and maintenance of various cancers ^16^.

This review article gives a short overview of the complex biology of IL-6 and explains how one cytokine can have extremely different biologic effects on different cells and in different physiologic states of the human body ^17^.

## The interleukin-6 receptor complex

**Figure 1.**
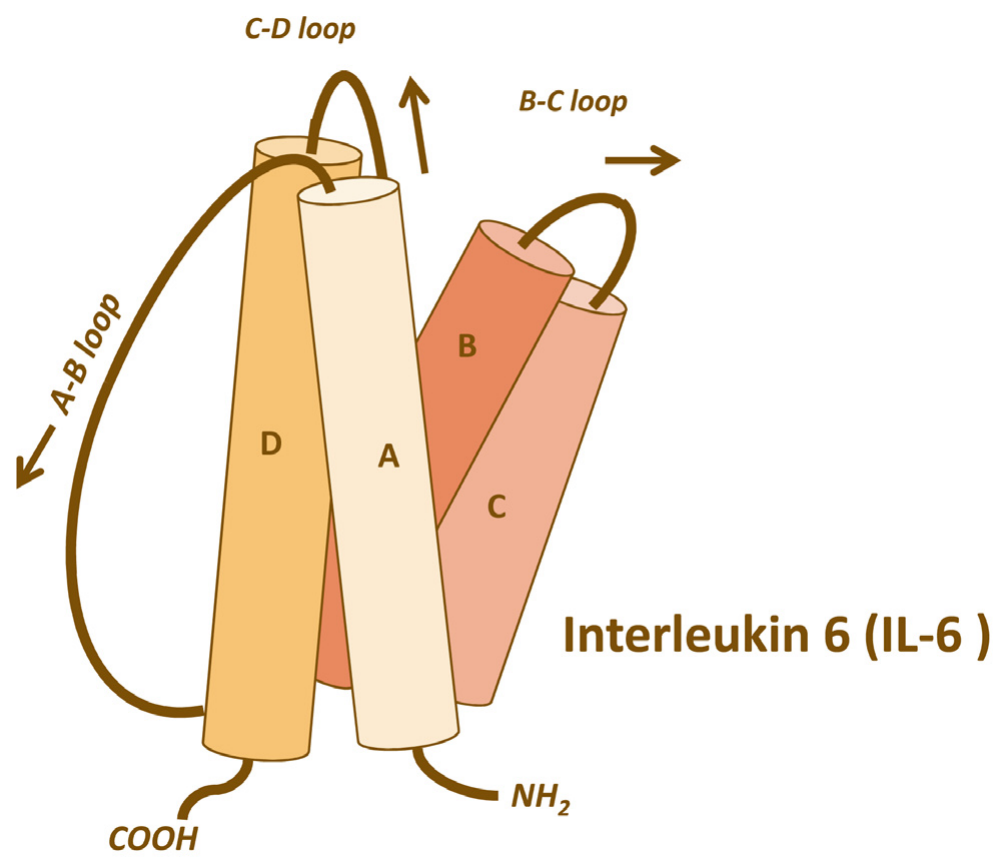
Four-helical topology of the interleukin-6 (IL-6) protein. IL-6 belongs to the family of four-helical cytokines. The figure shows the four helices with the connecting loops. The A–B and the C–D loops are long enough to reach the length of a helix, whereas the B–C loop is short. Consequently, IL-6 has an up-up-down-down topology, meaning that helices A and B point upwards, whereas helices C and D point downwards. This topology is common to most cytokines such as IL-2, IL-4, IL-7, IL-11, IL-15, leukaemia inhibitory factor, oncostatin M, growth hormone, leptin, and many others.

**Figure 2.**
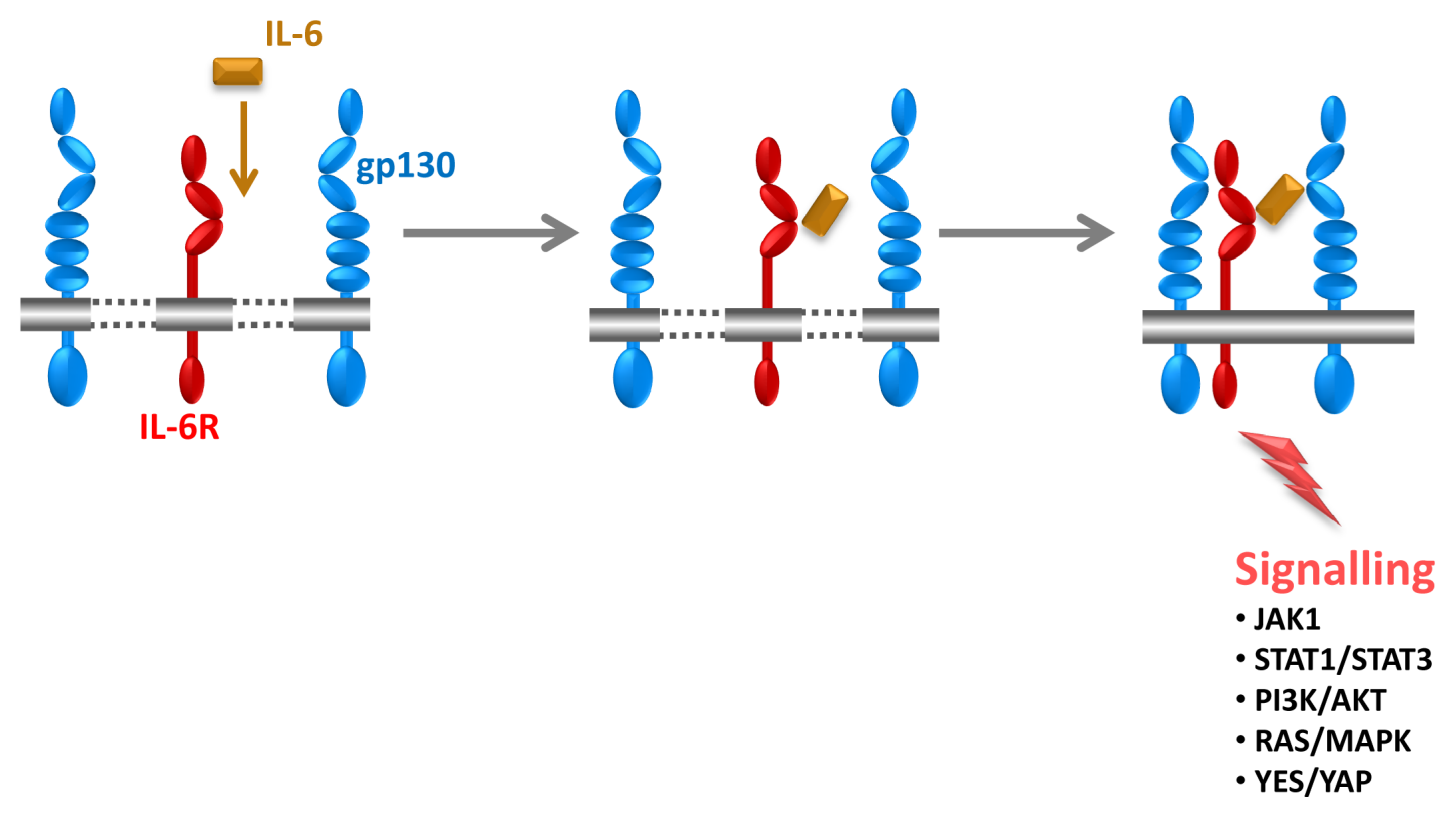
Stimulation of target cells by interleukin-6 (IL-6). IL-6 (orange) first binds to the IL-6 receptor (IL-6R) (red). The complex of IL-6 and IL-6R associates with glycoprotein 130 kDa (gp130) (blue), which dimerises and leads to intracellular signalling. It is important to note that IL-6 and IL-6R alone exhibit no measurable affinity to gp130. Only the complex of IL-6 and IL-6R binds to and activates gp130. Therefore, IL-6 cannot stimulate cells that do not express IL-6R. Signalling occurs via the signal transducer and activator of transcription (STAT) 1/STAT3, Yamaguchi sarcoma viral oncogene homolog (YES)/YES-associated protein (YAP), phosphoinositide-3 kinase (PI3K)/AKT, and rat sarcoma proto oncogene (RAS)/mitogen-activated protein kinase (MAPK) pathways. JAK, Janus kinase.

The four-helical cytokine IL-6 ( Figure 1) on cells binds to a membrane-bound IL-6 receptor (IL-6R), and the complex of IL-6 and IL-6R associates with a second receptor protein, glycoprotein 130 kDa (gp130), which dimerises and initiates intracellular signalling via the Janus kinase (JAK)/signal transducer and activator of transcription (STAT) and rat sarcoma proto oncogene (ras)/mitogen-activated protein kinase and phosphoinositide-3 kinase pathways ( Figure 2) ^18^. Importantly, IL-6 exhibits only a measurable affinity to the IL-6R but not to gp130, and the IL-6R does not bind on its own to gp130. It is only the complex of IL-6 and IL-6R that binds to gp130 and induces its dimerisation ( Figure 2). All cells in the body express gp130, but only a few cells such as hepatocytes and some leukocytes express IL-6R. It follows that cells that express only gp130 but not IL-6R cannot be stimulated by IL-6 ^1^.

Noteworthy, gp130 is a component of the receptor complexes of the so-called gp130 cytokine family, which besides IL-6 comprises IL-11, ciliary neurotrophic factor (CNTF), cardiotrophin-1 (CT-1), cardiotrophin-like cytokine (CLC), leukaemia inhibitory factor (LIF), oncostatin M (OSM), and IL-27. For details, please refer to recent reviews ^19, 20^.

**Figure 3.**
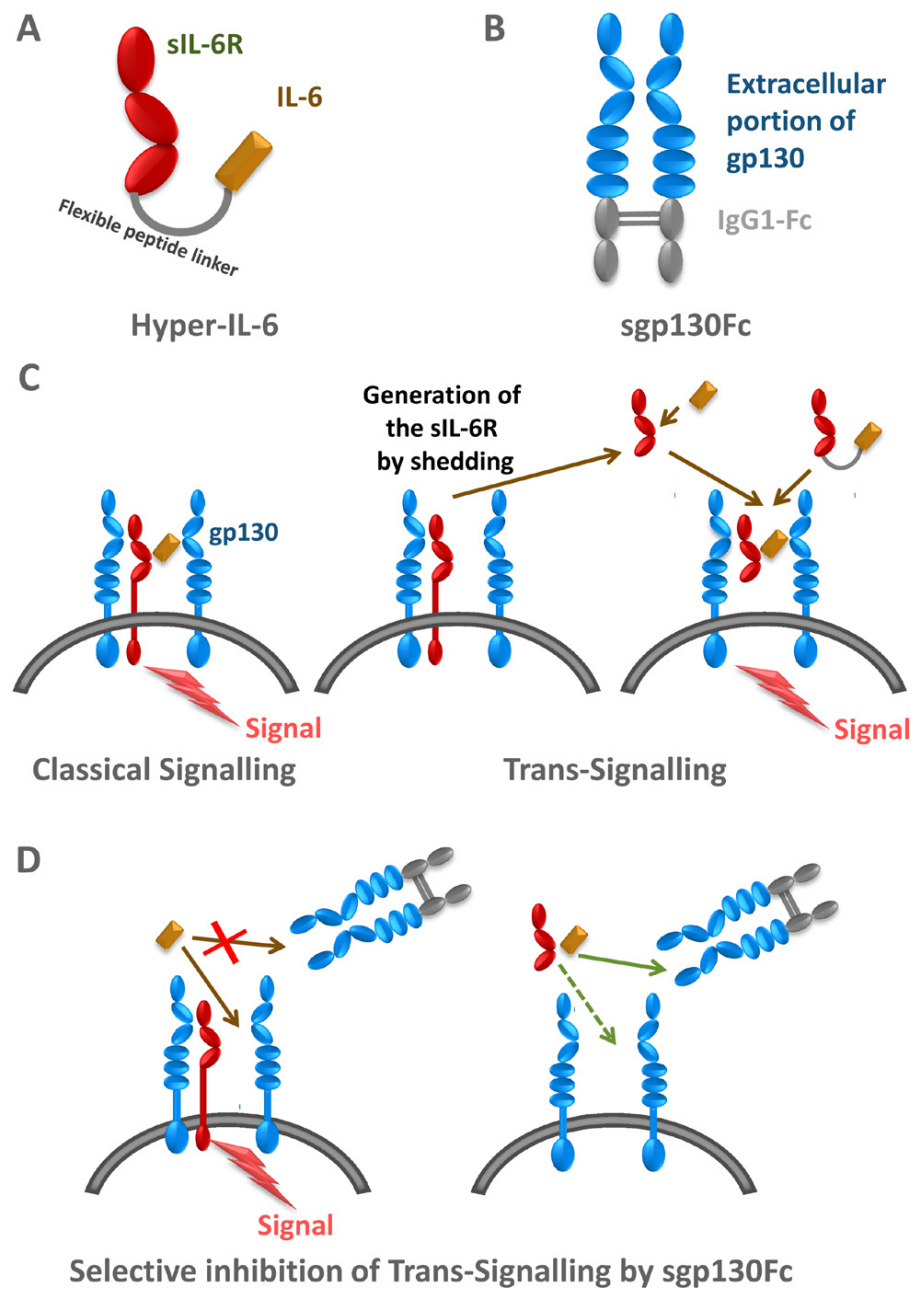
Designer proteins to probe for modes of interleukin-6 (IL-6) signalling. ( **A**) Hyper-IL-6 is a fusion protein between IL-6 and soluble IL-6 receptor (sIL-6R). ( **B**) sgp130Fc is a fusion protein of the extracellular portion of glycoprotein 130 kDa (gp130) and the constant part of a human immunoglobulin G1 (IgG1) antibody. ( **C**) IL-6 can signal via the membrane-bound IL-6R (classical signalling) and via the sIL-6R (trans-signalling). Hyper-IL-6 can be used to mimic IL-6 trans-signalling. ( **B**) The sg130Fc protein does not interfere with classical IL-6 signalling, but it specifically blocks IL-6 trans-signalling.

It has, however, been noticed that the membrane-bound IL-6R can be cleaved by the membrane-bound metalloprotease a disintegrin and metalloprotease 17 (ADAM17) to generate a soluble IL-6R (sIL-6R) ^21^. To a minor extent, the human—but not the murine—sIL-6R can be generated by translation from a differentially spliced mRNA ^22^. Intriguingly, the sIL-6R can still bind IL-6, and the complex of IL-6 and sIL-6R can associate with gp130 and induce signalling, even on cells that lack the membrane-bound IL-6R ^23^. This process has been named IL-6 trans-signalling ( Figure 3) ^24^. Strikingly, following this paradigm, IL-6 can, in the presence of sIL-6R, stimulate any cell in the body since all cells express gp130 ^17^.

Interestingly, most IL-6R-expressing cells including hepatocytes express far more gp130 than IL-6R molecules. Therefore, stimulation of such cells with IL-6 alone will only lead to engagement of few gp130 molecules, whereas stimulation with the complex of IL-6 and sIL-6R will stimulate all cellular gp130 proteins. A threshold for a given response might not be reached with IL-6 stimulation but only with stimulation of all gp130 molecules via IL-6 trans-signalling. This might be an explanation for the observed differences in signalling between trans-signalling and classical signalling that lead to different phenotypes ^25^.

## Molecular tools to elucidate the functions of interleukin-6

The concept of IL-6 trans-signalling has been corroborated by the use of two designer proteins. The first such protein consists of IL-6 covalently fused to the sIL-6R via a 40 Å flexible peptide linker, which allowed the placement of IL-6 at the correct distance to reach the IL-6 binding site of the sIL-6R. This protein was called Hyper-IL-6 ( Figure 3A) ^26^. This protein was shown to stimulate gp130-expressing cells *in vitro* and *in vivo*, and it was shown that liver regeneration ^27^, stimulation of neural cells ^28^, and expansion of hematopoietic cells ^29^ was far more efficient in the presence of Hyper-IL-6 as compared to IL-6 alone ^30^.

**Figure 4.**
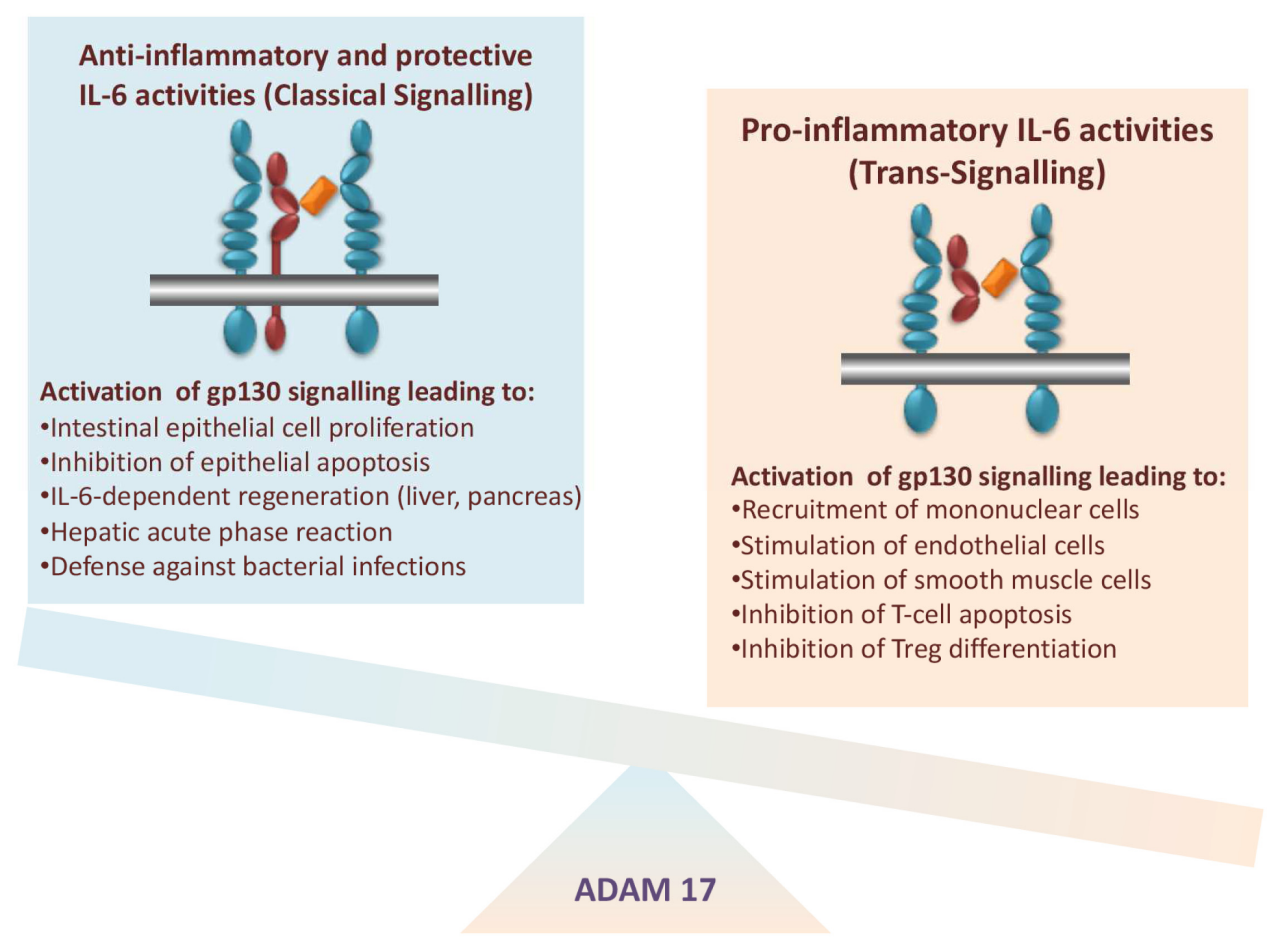
Pro- and anti-inflammatory activities of interleukin-6 (IL-6). Left, anti-inflammatory and protective activities of the cytokine IL-6 are associated with signalling via the membrane-bound IL-6 receptor (IL-6R). Right, pro-inflammatory activities of the cytokine IL-6 are associated with signalling via the soluble IL-6R (sIL-6R). The membrane-bound metalloprotease a disintegrin and metalloprotease 17 (ADAM17) orchestrates the pro- and anti-inflammatory activities of IL-6. Treg, regulatory T cell.

While Hyper-IL-6 demonstrated only the biologic potential of IL-6 trans-signalling, these experiments did not prove that this process occurred *in vivo*. A second soluble protein was designed, which consisted of the entire extracellular portion of gp130 covalently fused to the Fc region of human IgG1 ( Figure 3B). The resulting protein, named soluble gp130Fc (sgp130Fc), turned out to exhibit similar properties as membrane-bound gp130: it did not bind IL-6 or IL-6R alone, but it bound with high affinity the complex of IL-6 and sIL-6R ^31, 32^. Consequently, the sgp130 protein *in vitro* and *in vivo* specifically inhibited IL-6 trans-signalling without compromising IL-6 signalling via the membrane-bound IL-6R, i.e. classic signalling ^32^. The sgp130Fc protein could be used to define IL-6-mediated biologic responses, which were dependent on classic or trans-signalling. This was accomplished by comparing the treatment of animals with sgp130Fc or with neutralising antibodies against IL-6 or IL-6R, which blocked all IL-6 signalling ( Figure 3C, D). Using animal models of human inflammatory diseases or inflammation-associated cancer, it turned out that autoimmune disorders and inflammation-associated cancers were mainly driven by IL-6 trans-signalling whereas regenerative and protective activities of IL-6 were mediated by classic IL-6 signalling via the membrane-bound IL-6R ( Figure 4) ^20^.

## Physiologic and pathophysiologic functions of interleukin-6

Under homeostatic conditions, IL-6 levels in the circulation are as low as 1–5 pg/ml, but during inflammatory states these levels can rise more than 1,000-fold, and under extreme conditions leading to sepsis IL-6 levels in the µg/ml range have been reported ^33^. IL-6 is produced by myeloid cells upon Toll-like receptor stimulation together with the cytokines IL-1β and tumor necrosis factor α (TNFα), which, via a feed-forward loop, lead to an immense amplification of IL-6 production during inflammatory conditions ^34^. There is perhaps no other protein in the human body whose level can go up by six orders of magnitude. This lets us conclude that IL-6 is the major alarm signal in the human body in response to infection, inflammation, and possibly cancer ^35^.

However, under normal conditions, IL-6 plays an important role in organ/cellular homeostasis. Mice in which the IL-6 gene has been ablated (IL-6 knockout mice) become obese late in life ^13^, cannot regenerate their liver upon hepatectomy ^36^, and show no signs of osteoporosis upon ovariectomy ^37^, indicating roles for IL-6 in body weight regulation, liver physiology, and bone metabolism. In pathophysiologic states, however, there are marked differences between IL-6 knockout mice and wild-type mice. IL-6 knockout mice are completely protected in animal models of rheumatoid arthritis ^38^ and multiple sclerosis ^39^, indicating a key role for IL-6 in these autoimmune disorders.

With the help of the sgp130Fc protein and of neutralising monoclonal antibodies, it was possible to selectively block IL-6 trans-signalling or to block all IL-6 signalling, respectively. Using this approach, it was shown that classic IL-6 signalling via the membrane-bound IL-6R was responsible for the defence of the body against bacteria ^40, 41^, intestinal regeneration upon polymicrobial sepsis ^42^, prevention of aortic rupture in animal models of abdominal aortic aneurysm ^43^, and healing of bone fractures ^44, 45^, indicating that these important processes are severely compromised under blockade of global IL-6 activity ^46^. It has been hypothesised that the same might apply for the treatment of COVID-19 patients ^46^ ( Figure 4).

Besides being the major alarm signal in the human body, IL-6 plays a dominant role in various types of cancer. One important reason could be that IL-6, via stimulation of the STAT3 pathway, is a prominent growth factor of many cancer cells. The following scenario has been worked out in pancreatic cancer ^47^. It was noted that in the Kras ^G12D^ model, the massive activation of the STAT3 pathway, which led to tumour progression, was induced by tumour-infiltrating myeloid cells, which stimulated the neoplastic cells via IL-6 trans-signalling ^47^. Selective blockade of this pathway by the sgp130Fc protein blocked progression of pancreatic intraepithelial neoplasias to pancreatic ductal adenocarcinomas ^47^, indicating a prominent role for IL-6 trans-signalling in the development of pancreatic cancer. In the murine APC ^min/+^ model of colon cancer, it was established that the genetic deletion of ADAM17, which is responsible for generating not only sIL-6R but also soluble TNFα and soluble ligands of the epidermal growth factor receptor (EGFR), resulted in completely abrogated tumour development ^16^. Moreover, the formation of neoplasias stimulated ADAM17 on macrophages, leading to EGFR ligand cleavage and subsequent EGFR stimulation. These macrophages now produced IL-6 and sIL-6R, which led to the outgrowth of the tumours. Again, selective blockade of the IL-6 trans-signalling pathway by the sgp130Fc protein blocked tumour development in the APC ^min/+^ model and an additional mouse model of colon cancer ^16^. This was highly reminiscent of a study in liver cancer, in which it was shown that the EGFR expressed in macrophages but not EGFR in hepatocytes was involved in the development of hepatocellular carcinoma ^48^. Apparently, macrophage activation may be an important step in the initiation and progression of tumours via the IL-6 trans-signalling pathway ^20^ ( Figure 4).

## Therapeutic targeting of interleukin-6 activity

Therapeutic targeting of the pro-inflammatory cytokine TNFα was introduced as an efficient strategy to treat patients with autoimmune disorders such as rheumatoid arthritis and inflammatory bowel disease ^49^. Subsequently, blockade of the biologic activity of the cytokine IL-6 was shown to be an efficient treatment for patients with rheumatoid arthritis and other autoimmune diseases ^2^, and it was shown that blocking IL-6 activity was more efficient than blocking TNFα in a monotherapy trial ^50^. Blockade of IL-6 activity with the IL-6R neutralising monoclonal antibody tocilizumab was also highly effective in the treatment of patients with CAR T cell-induced severe cytokine release syndrome ^51^. In patients with severe COVID-19 disease, the administration of tocilizumab resulted in a marked improvement of the condition in the majority of patients: the fever subsided, C-reactive protein decreased, and oxygen intake could be lowered. No obvious adverse reactions were observed. These preliminary data indicated that tocilizumab is a candidate for effective treatment of COVID-19 patients ^5, 52^. Interestingly, treatment of COVID-19 patients with the IL-6R neutralising monoclonal antibody sarilumab resulted in no significant difference in clinical improvement and mortality ^53^.

## Summary

The discovery that the pro-inflammatory activities of IL-6 are mediated by IL-6 trans-signalling whereas the protective and regenerative activities of IL-6 rely on classic signalling via the membrane-bound IL-6R suggested that the sgp130Fc protein might be an ideal candidate for a more specific mode of cytokine blockade as opposed to global cytokine inhibition ^20^. It was shown in appropriate animal models that blockade of IL-6 trans-signalling was indeed superior to global IL-6 blockade in a bone healing model ^44, 45^, in a sepsis model ^42^, in abdominal aortic aneurysm models ^43^, and in bacterial infection models ^40, 41^. The sgp130Fc protein was expressed and purified according to GMP regulations. Phase I clinical trials were successfully performed with healthy individuals, and a phase II clinical trial is presently ongoing in patients with inflammatory bowel disease ^54^. The future will tell whether this elegant therapeutic approach, which was successfully tested in many animal models, leads to a novel paradigm in cytokine-blocking therapies in patients with autoimmune disorders ^46^. Similarly, blockade of trans-signalling while leaving classical signalling intact may prove to be beneficial for patients experiencing “cytokine storms” from COVID-19 or CAR T-cell therapies. Finally, we suggest that malignancies promoted by high levels of trans-signalling could be contained by this therapeutic modality.

## Abbreviations

ADAM17, a disintegrin and metalloprotease 17; EGFR, epidermal growth factor receptor; gp130, glycoprotein 130 kDa; IL-6, interleukin-6; IL-6R, interleukin-6 receptor; ras, rat sarcoma proto oncogene; sgp130Fc, soluble gp130-Fc fusion protein, which under the name of olamkicept is in phase II clinical trials; sIL-6R, soluble IL-6R; STAT, signal transducer and activator of transcription; TNFα, tumor necrosis factor α; YAP, YES-associated protein; YES, Yamaguchi sarcoma viral oncogene homolog.
